# St. Thomas's Hospital

**Published:** 1889-10-12

**Authors:** 


### ST. GEORGE'S HOSPITAL.

### ST. THOMAS'S HOSPITAL.

Mr. William Anderson delivered the introductory address at St. Thomas's. The lecture consisted of a review of surgery from the earliest period. After a reference to the rise of Greek surgery, and its transmission by the Arabs, a picture was drawn of an European surgeon of the close of the mediæval period. An account was then given of the origin and progress of barber-surgeons. With the sixteenth century a new era opened, the chief advances being in the rational treatment of gunshot wounds, the power of controlling hæmorrhage, and the relief of strangulated hernia. The seventeenth century was illuminated by Harvey's discovery of the circulation of the blood, and the researches of Acelli and Pecquel on the lymphatic vessels. The treatment of gunshot wounds also made progress under Wiseman and Magati. But surgical education remained in a low state, excepting in Italy, where a system of clinical instruction was established. In the eighteenth century the scientific teaching of anatomy and surgery made great strides under Hunter, Petit, Pott, and others, and various important medical schools were founded. The present century was described as one of phenomenal progress, owing to the extension of medical education, the great development of the medical press, and the multiplication of scientific appliances.

